# Second month sputum smear as a predictor of tuberculosis treatment outcomes in Brazil

**DOI:** 10.1186/s13104-018-3522-3

**Published:** 2018-06-28

**Authors:** Maria do Socorro Nantua Evangelista, Rosalia Maia, João Paulo Toledo, Ricardo Gadelha de Abreu, José Uereles Braga, Draurio Barreira, Anete Trajman

**Affiliations:** 1 Brazil, Ministry of Health, National Tuberculosis Program, Federal District, Brasília, Brazil; 2 University of Brasilia (UnB), Federal District, Brasília, Brazil; 3 SHIS QI 27 Conj. 10 Casa 8. Lago Sul, Brasília, 71675-100 Brazil; 4 University of the State of Rio de Janeiro (UERJ), Rio De Janeiro, RJ Brazil; 5 UNITAID-WHO, Geneva, Switzerland; 6 Federal University of Rio de Janeiro (UFRJ), Rio De Janeiro, RJ Brazil; 7 McGill University, Montreal, QC Canada

**Keywords:** Acid-fast bacilli, Predictive value, Pulmonary tuberculosis, Sputum smear microscopy, Treatment outcomes

## Abstract

**Objective:**

The value of sputum smear microscopy (SSM) after 2 months of treatment in the management of pulmonary tuberculosis is controversial. We analysed second month-SSM conversion as a predictor of treatment success in Brazil.

**Results:**

Overall successful outcome rate was 89.4%. The predictive value of second month-SSM conversion for successful outcomes was 85.2% 72,479/85,118), while the predictive value of non-conversion for unfavourable outcomes was 26.9% (2712/10,071). Unfavourable treatment outcomes were twice more likely among patients who did not convert (adjusted OR = 2.06; 1.97–2.16).

## Introduction

Adverse outcomes of tuberculosis (TB) treatment still hamper the control of the disease worldwide despite the high efficacy of 6 month-regimen recommended by the World Health Organization [1]. TB treatment follow-up usually consists of monthly sputum smear microscopy (SSM) and an end-of-treatment chest X-ray [1, 2]. The bacillary burden usually decreases steadily during treatment and by the end of the second month of treatment, when the intensive phase of treatment is concluded, SSM is expected to be negative in most cases [2, 3]. The negativation of a previously positive test is known as SSM conversion. SSM non-conversion after 2 months of treatment is recognized as a predictor of unfavourable outcomes [4–8], including drug-resistance [8–11]. Conversely, failure is unlikely if SSM during all months of treatment are negative [7, 12, 13].

The Brazilian guidelines [14] recommend monthly SSM for smear-positive TB follow-up plus culture with drug susceptibility test (DST) if SSM conversion is not observed after 2 months of treatment. However, the predictive value of SSM conversion for treatment outcomes has not been carried-out in Brazil [14, 15]. The aim of this study was to evaluate the positive predictive value of second month-SSM conversion for successful treatment outcomes in Brazil as well as of non-conversion for unfavourable outcomes.

## Main text

### Study design and population

A retrospective cohort study based on TB data recorded in the Brazilian National Surveillance System (SINAN) [14] was conducted. Data gathered included patients’ sociodemographic characteristics, their SSM results during follow-up and treatment outcomes up to 9 months after treatment initiation.

### Outcome definitions

Outcomes were as reported in the notification system. The following categories exist as outcomes according to the Brazilian guidelines [15]: (1) cure, defined as an individual who presents at least two negative SSM of which one at the end of treatment (5th or 6th month); (2) treatment completion, defined when there is no clinical or radiological evidence of failure; (3) death from TB; (4) death from non-TB causes; (5) loss to follow up, i.e., a patient who missed a scheduled follow up visit for at least 30 days; (6) failure, i.e., a positive SSM result at the end of treatment, SSM with 2+ or 3+ at the 4th month of treatment or a positive SSM at the 4th month of treatment after initial conversion; (7) change of diagnosis and (8) transferred-out.

We further classified these outcomes as successful (cure or treatment completed) or unfavourable (death from any cause, loss to follow up or failure) [16, 17].

### Study population

New smear-positive pulmonary TB adults (> 14 years) notified from January 2007 to December 2012 in any Brazilian municipality were eligible. Patients transferred-out and those whose diagnosis was changed were excluded, since true outcomes or diagnosis were uncertain. For the main analysis, we further excluded patients whose status of SSM at the second month was unknown (not done/no results/not informed).

### Analyses

We compared characteristics of initially included patients according to availability of second month SSM results, to check for selection bias.

The main analysis consisted of evaluating the positive predictive value and its exact 95% confidence intervals (95% CI) of a positive second month-SSM (non-conversion) for unfavourable treatment outcomes and of a negative second month-SSM (conversion) for successful outcomes. This was calculated through the proportion of patients with a positive second month SSM out of those who had unfavourable outcomes and the proportion of those with a negative second month SSM who had successful outcomes, respectively. Simple and multiple logistic regression models were used to calculate the odds ratios (OR) and their 95% CI to evaluate the independent effect of the second month-SSM result on unfavourable treatment outcomes, adjusted for sociodemographic variables. Analyses were performed using the SPSS^®^ package, version 20.0 (IBM Inc., Armonk, NY, USA).

## Results

**Fig. 1:**
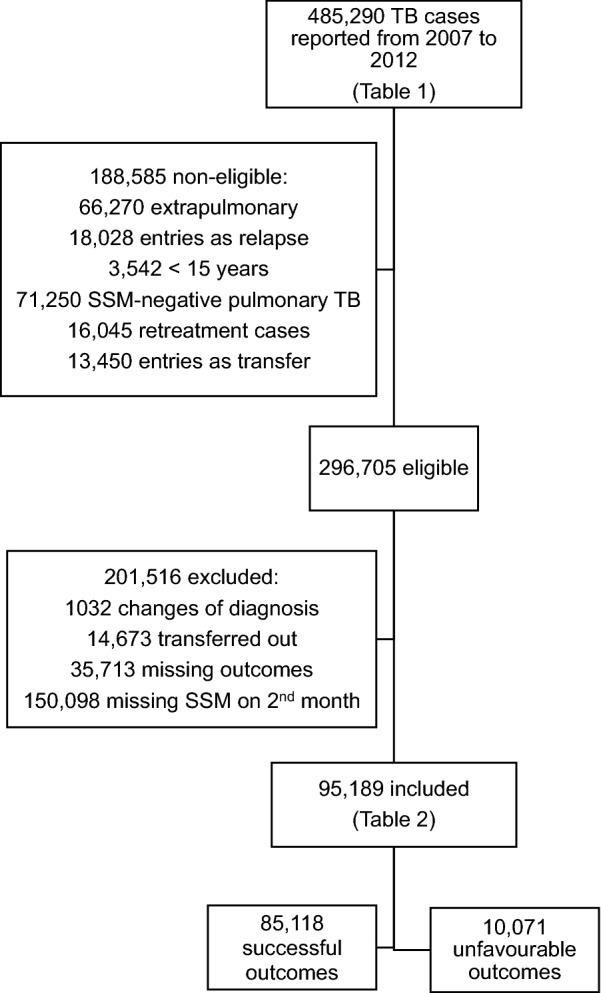
Flowchart representing eligible, excluded and included patients and their treatment outcomes

A total of 485,290 TB cases were notified from 2007 to 2012, of which 188,585 were not eligible and 201,516 were excluded. The remaining 95,189 were analysed (Fig. 1). Sociodemographic characteristics and second month-SSM results of included versus non-eligible and excluded patients were similar (Table 1). Most of included patients (Table 1) presented SSM conversion at the second month (83.9%), were male (67.2%), aged 15–54 (80.6%), had mixed race (42.4%) and less than 9 years of study (47.9%). Overall successful treatment rate was 89.4%. Missing data were more common among excluded patients and those with successful treatment.

**Table 1:** Characteristics of included and excluded tuberculosis new cases aged 15 or over notified to the Brazilian National Reporting System, 2007–2012

Variables	Included patients^a^ n = 95,189 n (%)	Excluded patients^b^ n = 390,101 n (%)
Smear conversion on 2nd month		
Yes	79,838 (83.9)	38,266 (9.8)
No	15,351 (16.1)	8260 (2.1)
Missing*	0	343,575 (88.1)
Sex		
Male	63,931 (67.2)	259,751 (66.6)
Female	31,255 (32.8)	130,318 (33.4)
Missing*	3 (0.0)	32 (0.0)
Age range (years)		
15–24	17,793 (18.7)	61,588 (15.8)
25–34	22,666 (23.8)	93,264 (23.9)
35–44	19,151 (20.1)	78,035 (20.0)
45–54	17,099 (18.0)	66,072 (16.9)
55–64	10,403 (10.9)	39,790 (10.2)
≥ 65	8032 (8.4)	34,618 (8.9)
Missing*	45 (0.0)	16,734 (4.3)
Race/colour		
White	30,903 (32.5)	130,071 (33.3)
Black	11,806 (12.4)	51,442 (13.2)
Asian	948 (1.0)	3651 (0.9)
Mixed race	40,394 (42.4)	152,319 (39.0)
Indigenous	1294 (1.4)	4046 (1.0)
Missing*	9844 (10.3)	48,572 (12.5)
Schooling		
Illiterate	5328 (5.6)	17,904 (4.6)
< 9 years	45,620 (47.9)	168,832 (43.3)
≥ 9 years	9941 (10.4)	43,671 (11.2)
Missing*	34,300 (36.0)	159,694 (40.9)

* Includes true missing data and SSM not performed

^a^Includes the following outcomes: cure, treatment completed, failure, relapse in this episode, loss to follow up, death from TB and other causes

^b^Includes the following outcomes: missing outcome, transferred out, change of diagnosis and missing smear conversion on 2nd month

The positive predictive value of the second month-SSM non-conversion for unfavourable outcomes was 26.9% (2712 out of 10,071), while the positive predictive value for successful outcomes among those who did convert second month SSM was 85.2% (72,479 out of 85,118).

Adjusted for sociodemographic characteristics, the odds for unfavourable outcomes were 2.06 higher (95% CI = 1.97–2.16) among those that had not converted at the second month of treatment (Table 2). Other variables significantly associated with higher odds for unfavourable outcomes were male gender (aOR = 1.50; 95% CI = 1.43–1.58); age 25–34 (aOR = 1.41; 95% CI = 1.07–1.22), age 35–44 (aOR = 1.13; 95% CI = 1.05–1.21), and age over 65 (aOR = 1.13; 95% CI = 1.04–1.23); black colour (aOR = 1.15; 95% CI = 1.08–1.23) and illiteracy (aOR = 1.60; 95% CI = 1.42–1.81). Indigenous populations (aOR = 0.69; 95% CI = 0.56–0.86) and mixed race (aOR = 0.91; 95% CI = 0.86–0.95) were inversely associated with unfavourable outcomes (Table 2).

**Table 2:** Treatment outcomes of adults (> 15 years) with smear-positive pulmonary tuberculosis notified to the Brazilian National Reporting System (2007–2012) according to their sociodemographic characteristics and second month SSM

Variables	Unfavourable^a^ n (%) 10,071 (10.6)	Successful^b^ n (%) 85,118 (89.4)	Crude OR^c^ (95% CI)	Adjusted OR^c^ (95% CI)
Smear conversion on 2nd month				
Yes	7359 (9.2)	72,479 (90.8)	1.0 (reference)	1.0 (reference)
No	2712 (17.7)	12,639 (82.3)	2.11 (2.01–2.22)	2.06 (1.97–2.16)
Sex				
Female	2465 (7.9)	28,790 (92.1)	1.0 (reference)	1.0 (reference)
Male	7606 (11.9)	56,325 (88.1)	1.58 (1.50–1.65)	1.50 (1.43–1.58)
Missing*	0	3 (100.0)	–	–
Age range				
15–24 years old	1688 (9.5)	16,105 (90.5)	1.0 (reference)	1.0 (reference)
25–34 years old	2510 (11.1)	20,156 (88.9)	1.19 (1.11–1.27)	1.41 (1.07–1.22)
35–44 years old	2229 (11.6)	16,922 (88.4)	1.26 (1.18–1.34)	1.13 (1.05–1.21)
45–54 years old	1771 (10.4)	15,328 (89.6)	1.10 (1.03–1.18)	0.96 (0.89–1.03)
55–64 years old	959 (9.2)	9444 (90.8)	0.97 (0.89–1.05)	0.85 (0.78–0.92)
65 years old or over	910 (11.3)	7122 (88.7)	1.12 (1.12–1.33)	1.13 (1.04–1.23)
Missing*	4 (8.9)	41 (91.9)	–	–
Race/color				
White	3327 (10.8)	27,576 (89.2)	1.0 (reference)	1.0 (reference)
Black	1428 (12.1)	10,378 (87.9)	1.14 (1.07–1.22)	1.15 (1.08–1.23)
Asian	100 (10.5)	848 (89.5)	0.98 (0.79–1.21)	1.02 (0.82–1.26)
Mixed race	3890 (9.6)	36,504 (90.4)	0.88 (0.84–0.93)	0.91 (0.86–0.95)
Indigenous	97 (7.5)	1197 (92.5)	0.67 (0.54–0.83)	0.69 (0.56–0.86)
Missing*	1229 (12.5)	8615 (87.5)	–	–
Education				
≥ 9 years of schooling	648 (6.5)	9293 (93.5)	1.0 (reference)	1.0 (reference)
< 9 years of schooling	4688 (10.3)	40,932 (89.7)	1.64 (1.51–1.80)	1.54 (1.42–1.68)
Illiterate	552 (10.4)	4776 (89.6)	1.66 (1.47–1.87)	1.60 (1.42–1.81)
Missing*	4183 (12.2)	30,117 (87.8)	–	–

*Includes true missing data and SSM not performed

^a^Successful = cure or treatment completion

^b^Unfavourable = loss to follow up, failure, death from tuberculosis or other causes, relapse of this episode, change of treatment

^c^Odds are for unfavourable outcomes

### Discussion and conclusions

In this retrospective analysis of a 5-year cohort of SSM-positive new TB cases in a high-TB burden country based on routine programmatic data, overall successful outcome rate was 89.4%. Having a positive SSM at the second month of treatment had a low predictive value (26.9%) for unfavourable outcomes while SSM conversion at this point had a high predictive value for successful outcomes (85.2%), although lower than reported in the literature [10, 13, 18]. However, the likelihood for unsuccessful outcomes was twice higher among those who did not convert the SSM by the second month. The low predictive value of second month-SSM was due to the high rates of successful treatment even among those who did not convert SSM by the second month of treatment.

The SSM non-conversion in the second month as a predictor of unfavourable outcomes has been a matter of debate in the literature [4, 5, 19, 20]. SSM conversion has been associated with cure/treatment completion [10, 13, 18]. However, non-conversion does not always indicate unfavourable outcomes because SSM has low sensitivity and low specificity to detect failure [21]. Dead bacilli, for example, are detected by SSM; only culture can distinguish dead from alive bacilli [22]. True positive second-month SSM results can be associated with comorbidities [5], extensive lesions and high bacterial load [23, 24], the so-called “difficult-to-treat” patients. Most of them will actually be cured at the end of treatment. However, irregularity of drug intake in the initial phase of treatment [7, 23] and the presence of resistant bacteria [10] can also be the reason for non-conversion at the second month and can result in unfavourable outcomes. Unfortunately, our study was based on programmatic data and we do not have, in the database, any information on the extension of the disease, treatment duration, or sputum culture results. Information on comorbidities is missing for most patients. Thus, they were not included in our analyses.

Other variables independently associated with higher odds for an unfavourable outcome are reported in the literature and were confirmed in our study: low educational level [25, 26], male gender [5, 10, 19, 27], older age [10, 20, 27] and black/mixed race [25]. Surprisingly, belonging to indigenous populations was inversely associated with unfavourable outcomes. Indigenous populations may be more difficult to reach and follow-up may be hampered [26, 28]. However, in Brazil, indigenous ethnicity is a formal indication for directly observed treatment [14] and special health services dedicated to this population receive special training [14, 15], which may explain this finding.

## Conclusions

The study included a large cohort of patients over 5 years and allowed to extract relevant information based on programmatic data, based on which decisions by the Ministry of Health are usually taken. While we conclude that the second month-SSM is a poor predictor of unfavourable outcomes, in the absence of a better predictor, we endorse the current recommendation to improve surveillance and perform culture and drug-susceptibility testing for patients with a positive second month-SSM. Other, more accurate early markers of poor prognosis are needed in order to trigger an alert to the treating health team.

## Limitations

This study has a few limitations. First, its retrospective design based on secondary data is subject to flaws. Missing data were a main concern. Missing information on follow-up smear results in Brazil has been reported previously [28, 29] and can be due both to incomplete data registration and to non-compliance with National Guidelines [14] to perform monthly SSM. Because missing data were not balanced among patients with different outcomes, our results should be interpreted with caution, since this can have resulted in bias. Bias can also have resulted from excluded patients, although their general characteristics were similar to the included ones. More missing data were expected in this group because despite the initial positive SSM, patients with other diseases either were excluded (such as non-tuberculous mycobacteria disease) or were transferred out or died from other causes, thus they had no follow-up SSM. More non-conversion was also expected since they were initially treated for TB but possibly had other diagnoses or were relapsed patients, who can have a delayed response to treatment. Finally, the database did not contain sufficient comorbidity, treatment duration and culture data.

## Abbreviations

TB: tuberculosis
SSM: sputum smear microscopy
CI: confidence interval
OR: odds ratio
DST: drug susceptibility test
SINAN: Brazilian National Surveillance System
